# Genetic parameters for milk, fat and protein yields in Murrah buffaloes (*Bubalus bubalis* Artiodactyla, Bovidae)

**DOI:** 10.1590/S1415-47572010005000005

**Published:** 2010-03-01

**Authors:** Rusbel Raúl Aspilcueta-Borquis, Roberta Cristina Sesana, Milthon Honorio Munoz Berrocal, Leonardo de Oliveira Seno, Annaiza Braga Bignardi, Lenira El Faro, Lucia Galvão de Albuquerque, Gregório Miguel Ferreira de Camargo, Humberto Tonhati

**Affiliations:** 1 Faculdade Ciências Agrárias e Veterinárias, Universidade Estadual Paulista Júlio de Mesquita Filho', Jaboticabal, SPBrazil; 2 Universidad Nacional Agraria de la Selva, Tingo MariaPerú; 3 Agência Paulista de Tecnologia dos Agronegócios, Ribeirão Preto, SPBrazil

**Keywords:** test-day model, accumulated productions, heritability, genetic correlations

## Abstract

The objective of the present study was to estimate genetic parameters for test-day milk, fat and protein yields and 305-day-yields in Murrah buffaloes. 4,757 complete lactations of Murrah buffaloes were analyzed. Co-variance components were estimated by the restricted maximum likelihood method. The models included additive direct genetic and permanent environmental effects as random effects, and the fixed effects of contemporary group, milking number and age of the cow at calving as linear and quadratic covariables. Contemporary groups were defined by herd-year-month of test for test-day yields and by herd-year-season of calving for 305-day yields. The heritability estimates obtained by two-trait analysis ranged from 0.15 to 0.24 for milk, 0.16 to 0.23 for protein and 0.13 to 0.22 for fat, yields. Genetic and phenotypic correlations were all positive. The observed population additive genetic variation indicated that selection might be an effective tool in changing population means in milk, fat and protein yields.

Milk, fat and protein yields are constantly monitored traits in herds integrating milk test programs. Test-day milk yield (TDM), defined as the total yield of a cow over a period of 24 h, replaces milk yield at 305 days of lactation (M305), as calculated by using formulas and extension factors (Tonhati *et al.* 2004). In addition, the application of TDM to the genetic evaluation of animals enables quantifying specific factors on each test-day that vary not only from animal to animal but also between the test-days themselves. It permits more reliable heritability estimates and a more accurate selection of the best individuals for future use in reproduction.

Several investigators have emphasized that environmental effects affecting certain test-days or lactation phases, such as management group, test-date, milking number, the herd itself, shape of the lactation curve, number of lactation days, preferential treatment of certain groups of cows and the specific effects of each cow on the test-day, which so far have been ignored in traditional models, can be adjusted by test-day models (Meyer *et al.*, 1989; Ptak and Schaeffer, 1993; Jamrozik and Schaeffer, 1997). Some of the advantages of test-day models (TDM) include the ability to account for the environmental effects of each test-day, the ability to model the trajectory of the lactation for individual genotype or groups of animals, and the possibility of genetic evaluation with a view to production persistency. (Jensen, 2001).

However, in order to choose which criteria should be adopted for genetic evaluation, accurate estimates of genetic variability and genetic correlation with P305 are important. Studies of Holstein and Gyr cattle and Murrah buffaloes have shown that genetic correlations between TDM and P305 are higher during the mid-lactation period when compared to the beginning and end of lactation (Ledic *et al.*, 2002; Ferreira *et al.*, 2003 and Hurtado-Lugo *et al.*, 2006).

There are little data on genetic parameters of milk yield and components during lactation in dairy buffaloes. Thus, the objective of the present study was to evaluate the possible application of TDM and its components in genetic evaluation of buffaloes, in the place of the traditional P305 model.

In the present study, 4,757 complete lactations of Murrah buffaloes, aged from 2 to 15 years, the daughters of 187 sires, and with calving records of the period 1985 to 2005, were analyzed. The animals belonged to 13 herds participating in the Buffalo Milk Test Program of the Animal Science Department of FCAV/UNESP, Jaboticabal, SP, Brazil.

Lactation recording was begun from the fifth day of lactation and truncated at the 305^th^. The first test was carried out up to 45 days after calving. Contemporary groups were defined according to herd-year-month of test, and consisted of 168 classes, with each group comprising at least four animals. A pedigree file containing 11,760 animals was used for all analyses.

(Co)variance components were estimated for milk, fat and protein yields, by finite dimensional test-day models dividing the lactation period into 9 time points (9 test days) as distinct traits, and using single- and two-trait analyses. (Co)variance components were estimated by the restricted maximum likelihood (REML) method using the MTDFREML statistical package (Boldman *et al.*, 1995).

The animal model can be represented as:

**y** = **X**ß + **Z***a* + **W***p* + *e*,

where: **y** is the vector of observations (milk, fat and protein yield), and **X**, **Z** and **W** are incidence matrices relating y to ß, *a* and *p*, the vectors of fixed effects (contemporary group and the cow's age as linear and quadratic effects) and additive genetic and permanent environmental random effects, respectively, and e is the vector of residual effects. This model comprises the following assumptions:



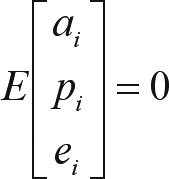
;



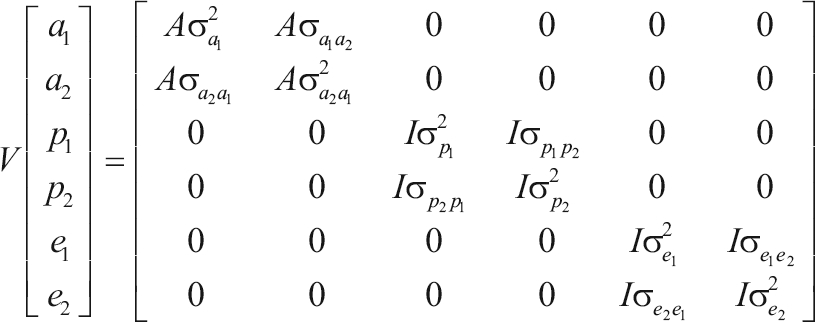


where *A* is the relationship matrix, *I* is an identity matrix, 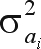, 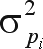 and 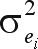 are additive genetic, permanent environmental and residual variances for trait *i* (*i* = 1, 2), respectively, and 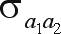, 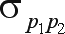 and 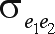 are additive genetic, permanent environmental and residual covariances between traits 1 and 2, respectively.

Expected genetic gain and correlated response to selection were obtained using estimates of heritability (*h*^2^), genetic correlations and phenotypic standard deviations. Selection of the best 5% males was examined, corresponding to a selection intensity factor of 2.06 (Lush, 1964). Females were randomly replaced in the herd, with selection intensity equal to zero. Thus, the mean selection intensity factor for all traits was 1.03. Expected direct and correlated responses to selection and the relative response efficiency were calculated by the usual selection index formulas, considering a progeny test with number of daughters per sire varying from 5 to 100.

Test-day milk-yield means revealed a typical lactation curve for Murrah buffaloes, starting with 8.12 kg and followed by a short increase until reaching the peak on the second test-day (8.61 kg), with a subsequent decrease until the end of lactation (Table 1). The P305 mean observed in this study (1,813.5 ± 697.40 kg) was higher than that obtained by Tonhati *et al.* (2000a, b) and Ramos *et al.* (2006) of 1,259.47 kg, 1,496.00 kg and 1,650 ± 687 kg, respectively. However, ours is similar to those reported by Malhado *et al.* (2007), 1,863.5 ± 677 kg, for this breed in Brazil and Shabade *et al.* (1993) for Murrah buffaloes in India (1,892.21 kg).

In fat and protein yield curves, the trend was similar to that observed for monthly milk yields, with higher means in early lactation, thereby suggesting a positive association between milk and its constituents. These trends are similar to those reported in the literature (Swalve, 1995) for milk, fat and protein yields in Holstein cows. Cumulative 305-day fat and protein yields were lower than those reported by Rosati and Van Vleck (2002), 196.9 ± 45.6 kg and 104.7 ± 21.7 kg, respectively, for Mediterranean buffaloes in Italy.

Additive genetic, permanent environmental and phenotypic variances estimates for test-day milk (TDM), fat (TDF) and protein yield (TDP) and cumulative 305-day milk (M305), fat (F305) and protein yields (PR305) obtained by two-trait analysis are shown in Table 2. Genetic variance estimates for TDM increased from the first (1.06 kg^2^) to the third (1.32 kg^2^) month, declining thereon until the end of lactation (0.52 kg^2^). The highest estimates were obtained for the third and fourth months of lactation. These results are similar to those reported for buffaloes by Hurtado-Lugo *et al.* (2006), who observed higher genetic additive variance around the fifth month. In general, higher genetic variance has been estimated around the fourth or fifth months of lactation for dairy cattle in Brazil (Ferreira *et al.*, 2003; Rodrigues *et al.*, 2005). Permanent environmental variance was higher around the first half of lactation. Phenotypic variance estimates showed the same trend of the additive genetic, increasing from the first to the third month of lactation with a posterior decrease (Table 3). The highest estimates of residual variance for milk were observed in the third and fourth months of lactation.

TDM heritability was higher in the third and fourth months of lactation due to higher genetic and lower residual variance (Table 2). Similar results have been reported by Jamrozik and Schaeffer (1997), Lidauer and Mäntysaari (1999) and Silvestre *et al.* (2005) in dairy cattle. These authors also suggested that selection for milk yield should be at the fourth or fifth month of lactation. Selection could be anticipated by promoting a reduction in the generation interval. In the present study, heritability for milk yield (0.12 to 0.24) was higher than that reported by Hurtado-Lugo *et al.* (2006) for Murrah buffaloes in Colombia (0.01 to 0.20).

For test-day fat yields (TDF), genetic variance increased from the first (3.02 x 10^-3^ kg^2^) to the fifth month (4.01 x 10^-3^ kg^2^), and then declined until the end of lactation (2.64 x 10^-3^ kg^2^). For dairy cattle, Swalve (1995) reported higher genetic variance at the beginning of lactation, with a subsequent oscillation throughout lactation. For permanent environmental variance (Table 2), there was an increase from the start to the fifth month of lactation, declining thereafter until the seventh month, thereupon increasing again during the last two months.

The estimated heritabilities (Table 2) for fat yields were moderate, the highest estimates being obtained for the sixth month of lactation (0.23). Studies with dairy cattle reported the same trend (Gengler *et al.*, 1997; Lidauer and Mäntysaari, 1999)

For test-day protein yields (TDP), genetic variance, as a whole, increased from the beginning (1.19 x 10^-3^ kg^2^) to the fourth and fifth months (2.12 x 10^-3^ kg^2^), thereupon decreasing until the end of lactation (1.21 x 10^-3^ kg^2^). A similar trend was reported by Swalve (1995). On the other hand, Silvestre *et al.* (2005) observed higher variances at the beginning of lactation, with a decrease till the fourth month and an increase thereafter. In the present study, the highest estimates of heritability were obtained in the fourth month of lactation (0.22), similar to that reported by Swalve (1995) and Lidauer and Mäntysaari (1999). However, Gengler *et al.* (1997) and Silvestre *et al.* (2005) found higher heritabilities for protein production in the ninth and seventh months of lactation, respectively.

The estimatives of selection efficiency and the direct response for accumulated yields (M305, F305 e Pr305), as well as the correlated response between accumulated and test-day yield (TDM, TDF e TDP), are presented in Table 3. It is possible to see that the direct responses to accumulated yields are higher than all the correlated answers in the three traits analyzed, thereby indicating that the use of accumulated responses as selection criteria could be a more efficient form of obtaining genetic gain.

The highest values for correlated response were observed among the yields in the second and sixth months of control for the traits analyzed. It is probable that these values were the outcome of the simultaneously high degree of heritability, as well as the high genetic correlation with the accumulated yields. The correlated response, according to the number of daughters per sire, can also be seen. As could be summarized, the greater the number of daughters per sire, the higher the estimate for correlated response. However, it is interesting to conceive that in present day dairy bubaline breeding, and with the small numbers of herds which compose the milk control program, a sire could take so long in generating 100 lactating daughters.

To appropriately use these studied traits, it is necessary to elaborate selection criteria based on an economic index evaluating the profitability of each trait for the breeder, and also indicate how selection must be done according to both the market and production.

The relative efficiency of selection for TDMs is greater at TDM5 and TDM6, which are the closest to M305. These results are similar to those described by Hurtado-Lugo *et al.* (2006) for Murrah buffaloes and Ferreira *et al.* (2003) for Holstein cows. However, in Gyr cattle, Ledic *et al.*, (2002), reported higher relative selection efficiency for TDM2, TDM3 and TDM4.

The highest correlated responses for 305-day fat yield, when using TDF yields as selection criteria, were obtained when TDF2 was adopted. In contrast, for 305-day protein yields, the highest correlated responses were observed on using TDP3, TDP4 and TDP5. Taken as a whole, the correlated genetic gain was higher for milk than for fat and protein yields.

Estimates of genetic correlation (Table 4) for TDM ranged from 0.54 to 1.00, with 64% of the correlations higher than 0.90, mainly between adjacent records. Melo (2005) reported estimates ranging from 0.64 to 1.00 in dairy cattle.

Lower genetic correlations were observed between TDM1 and the last two test-days, probably due to the low lactation persistency in buffaloes. All phenotypic correlations were positive, these ranging from 0.21 (between TDM1 and TDM9) to 0.69 (between TDM4 and TDM5). Phenotypic correlations gradually decreased with the increasing distance between test-days, reaching 89% lower than 0.60.

Estimates of genetic correlation ranged from 0.46 to 0.99 for TDF and from 0.47 to 0.94 for TDP. Most of the estimates were high, almost reaching one. In addition, higher estimates were obtained when test-day yields were closer together. Lower genetic correlations were observed between test-day yields during the first half of lactation and the production of the ninth test-day. A possible explanation could be that lactation in buffaloes tends to be of shorter duration and less persistent than in dairy cattle. Gengler *et al.* (1997) and Silvestre *et al.* (2005), on estimating genetic parameters in dairy cows, also observed high genetic correlations between adjacent test-days and low correlations between those distant.

The phenotypic correlations for TDF were positive and ranged from 0.36 (between TDF1 and TDF9) to 0.76 (between TDF1 and TDF2), and for TDP the range was from 0.35 to 0.71. Similar results were reported by Gengler *et al.* (1997) and Silvestre *et al.* (2005) for dairy cattle.

Regarding genetic correlations between different test-days for milk, fat and protein (not shown), lower correlations were observed between milk and fat yields and between fat and protein yields when compared to that between milk and protein yields. Overall, all the estimates were positive, thereby inferring desirable association between milk and its constituents. Similar results were obtained for phenotypic correlations.

In conclusion, the higher estimates for genetic variance in the first four test-days suggested that these test-day yields could be used as a selection criterion, reducing the generation interval. A greater response to selection for cumulative 305-day milk, fat and protein yields might be obtained by direct selection for these traits. The use of milk, fat and protein yields on any test-day as selection criteria will result in a correlated response for all other test days, as well as for 305-day yields. A higher correlated response for 305-day yields might be obtained when using mid-lactation (3rd to 6th test day) records as selection criteria.

## Figures and Tables

**Table 1:** Number of observations (N), observed means (kg), standard deviation (SD, kg) and coefficients of variation (CV) for monthly test-day milk, fat and protein yields (TDM1 to TDM9) and cumulative 305-day milk, fat and protein yields (P305) in Murrah buffaloes.

	Yield										
	Milk				Fat				Protein		
Trait	N	Mean ± SD	CV%		N	Mean ± SD	CV%		N	Mean ± SD	CV%
TDM1	6153	8.12 ± 3.14	36.49		825	0.44 ± 0.16	36.49		821	0.32 ± 0.11	33.90
TDM2	6175	8.61 ± 3.17	34.95		936	0.47 ± 0.16	33.23		936	0.32 ± 0.10	31.27
TDM3	6032	8.30 ± 3.10	35.49		885	0.46 ± 0.15	33.34		885	0.30 ± 0.10	32.94
TDM4	5862	7.74 ± 2.92	35.88		888	0.46 ± 0.17	36.10		887	0.29 ± 0.10	36.54
TDM5	5580	7.17 ± 2.72	36.65		885	0.44 ± 0.15	34.04		885	0.27 ± 0.10	36.61
TDM6	5211	6.56 ± 2.55	37.50		784	0.42 ± 0.16	36.32		784	0.25 ± 0.11	43.91
TDM7	4816	5.94 ± 2.30	37.91		710	0.39 ± 0.14	36.62		709	0.23 ± 0.09	39.91
TDM8	4075	5.43 ± 2.17	39.25		593	0.40 ± 0.15	37.76		593	0.23 ± 0.09	40.12
TDM9	3710	4.76 ± 1.96	39.83		502	0.39 ± 0.15	37.95		502	0.22 ± 0.09	40.87
P305	4757	1,813 ± 697	38.37		525	118.3 ± 29.5	37.02		597	81.6 ± 17.7	39.18

**Table 2:** Estimates of additive genetic ( ${\hat{\sigma }}_{a}^{2}$), permanent environmental ( ${\hat{\sigma }}_{ap}^{2}$) and ( ${\hat{\sigma }}_{p}^{2}$) phenotypic variance (kg^2^), estimates of heritability ( ${\hat{h}}^{2}$), and genetic correlations (*r*_*g*_), for milk, fat and protein yields obtained by two-trait analysis.

	${\hat{\sigma }}_{a}^{2}$	${\hat{\sigma }}_{ap}^{2}$	${\hat{\sigma }}_{p}^{2}$	*r*_*g*_	${\hat{h}}^{2}$
Milk					
TDM1	1.06	1.24	5.49	0.99	0.19
TDM2	1.13	1.79	5.70	0.89	0.20
TDM3	1.32	1.66	5.59	0.91	0.24
TDM4	1.23	1.48	5.24	0.97	0.23
TDM5	1.10	1.50	4.89	1.00	0.22
TDM6	0.92	1.29	4.39	0.99	0.21
TDM7	0.61	1.29	3.84	0.98	0.16
TDM8	0.57	1.16	3.86	0.94	0.15
TDM9	0.52	1.26	3.49	0.95	0.15
M305	78681.16	122543.63	299971.54		0.26

Fat					
TDF1*	3.02	2.32	16.34	0.82	0.19
TDF2*	3.21	2.43	16.44	0.88	0.20
TDF3*	3.71	2.30	17.34	0.78	0.21
TDF4*	3.98	2.96	18.52	0.80	0.21
TDF5*	4.01	2.93	18.63	0.76	0.21
TDF6*	3.66	1.81	16.04	0.71	0.23
TDF7*	3.01	1.78	13.86	0.74	0.22
TDF8*	2.88	2.04	16.32	0.69	0.20
TDF9*	2.64	2.14	16.44	0.79	0.16
F305	107.48	32.37	507.56		0.21

Protein					
TDP1*	1.19	1.70	8.01	0.68	0.15
TDP2*	1.48	2.48	8.90	0.78	0.17
TDP3*	2.09	2.93	10.01	0.82	0.21
TDP4*	2.12	3.02	9.73	0.80	0.22
TDP5*	2.12	3.62	10.21	0.82	0.21
TDP6*	2.03	3.48	10.20	0.76	0.20
TDP7*	1.58	3.43	9.63	0.72	0.16
TDP8*	1.41	3.35	8.88	0.81	0.16
TDP9*	1.21	3.49	9.48	0.77	0.13
Pr305	33.,33	19.14	185.9		0.18

*Variance multiplied by 1000.

**Table 3:** Expected direct response to selection for 305-days milk (M305), fat (F305) and protein (Pr305) yields, and respective correlated responses and relative selection efficiency selecting for test-day yields (milk, fat and protein).

	Correlated response (kg)					Relative selection efficiency (%)			
Trait	Number of daughters by sire					Number of daughters by sire			
	5	20	50	100		5	20	50	100
	Milk								
M305^(^*^)^	142.46	213.93	247.19	262.27		100	100	100	100
TDM1	124.06	196.23	234.61	253.45		87.08	91.73	94.91	96.64
TDM2	113.95	178.78	212.51	228.85		79.99	83.57	85.97	87.26
TDM3	125.55	191.14	222.74	237.34		88.13	89.35	90.11	90.49
TDM4	131.55	201.71	236.12	252.20		92.34	94.29	95.52	96.16
TDM5	133.18	205.72	241.99	259.12		93.48	96.17	97.90	98.80
TDM6	129.34	201.34	238.04	255.59		90.79	94.11	96.30	97.45
TDM7	114.14	185.33	225.96	246.86		80.12	86.63	91.41	94.12
TDM8	106.47	174.49	214.34	235.21		74.74	81.57	86.71	89.68
TDM9	107.60	176.35	216.62	237.71		75.53	82.43	87.63	90.64

	Fat								
F305*	152.70	237.69	281.02	301.74		100	100	100	100
TDF1**	120.10	189.97	227.12	245.35		78.65	79.92	80.82	81.31
TDF2**	131.68	206.60	245.59	264.47		86.24	86.92	87.39	87.65
TDF3**	119.11	185.40	219.20	235.36		78.00	78.00	78.00	78.00
TDF4**	122.16	190.15	224.82	241.39		80.00	80.00	80.00	80.00
TDF5**	116.05	180.65	213.58	229.32		76.00	76.00	76.00	76.00
TDF6**	112.54	172.56	202.00	215.76		73.70	72.60	71.88	71.50
TDF7**	115.18	177.93	209.29	224.11		75.43	74.86	74.47	74.27
TDF8**	103.25	161.99	192.56	207.37		67.62	68.15	68.52	68.72
TDF9**	107.54	174.61	212.89	232.58		70.43	73.46	75.76	77.08

	Protein								
Pr305*	79.72	127.16	152.97	165.82		100	100	100	100
TDP1**	50.13	82.16	100.92	110.74		63	65	65.97	66.79
TDP2**	60.69	97.65	118.23	128.64		76.13	76.79	77.29	77.58
TDP3**	69.73	108.54	128.32	137.79		87.47	85.35	83.89	83.09
TDP4**	69.34	107.12	126.00	134.92		86.98	84.24	82.37	81.37
TDP5**	69.73	108.54	128.32	137.79		87.47	85.35	83.89	83.09
TDP6**	63.33	99.36	118.11	127.19		79.44	78.14	77.21	76.71
TDP7**	54.58	88.62	108.05	118.04		68.47	69.69	70.64	71.19
TDP8**	61.40	99.70	121.55	132.80		77.02	78.40	79.47	80.09
TDP9**	53.31	89.11	111.30	123.40		66.87	70.08	72.76	74.42

*Expected direct genetic gain. **Correlated response multiplied by 10^3^.

**Table 4:** Heritability (diagonal), genetic correlations (above the diagonal) and phenotypic correlations (below the diagonal) estimated by two-trait-analysis for milk, fat and protein test-day yields.

		Milk								
		1	2	3	4	5	6	7	8	9
	1	0.18	0.96	0.97	0.91	0.86	0.78	0.79	0.66	0.54
	2	0.55	0.20	0.98	0.93	0.87	0.89	0.82	0.60	0.84
	3	0.51	0.64	0.24	0.94	0.95	0.94	0.90	0.69	0.89
	4	0.48	0.59	0.66	0.23	0.91	0.98	0.97	0.85	0.90
Milk	5	0.42	0.53	0.58	0.69	0.22	0.98	0.94	0.95	0.94
	6	0.36	0.46	0.51	0.52	0.64	0.19	0.99	0.96	0.94
	7	0.26	0.39	0.46	0.48	0.52	0.56	0.15	0.99	1.00
	8	0.25	0.30	0.36	0.39	0.43	0.44	0.52	0.13	0.98
	9	0.21	0.27	0.29	0.31	0.33	0.34	0.40	0.46	0.13

		Fat								
	1	0.18	0.96	0.86	0.75	0.68	0.55	0.48	0.49	0.46
	2	0.76	0.19	0.95	0.86	0.76	0.69	0.63	0.56	0.54
	3	0.71	0.71	0.21	0.96	0.89	0.80	0.74	0.67	0.54
	4	0.63	0.65	0.68	0.21	0.97	0.89	0.83	0.76	0.65
Fat	5	0.48	0.59	0.62	0.68	0.21	0.96	0.91	0.87	0.81
	6	0.52	0.54	0.55	0.61	0.67	0.23	0.99	0.90	0.83
	7	0.57	0.48	0.51	0.56	0.62	0.64	0.22	0.96	0.89
	8	0.43	0.46	0.48	0.50	0.54	0.59	0.68	0.18	0.93
	9	0.36	0.41	0.43	0.46	0.49	0.57	0.61	0.67	0.15

		Protein								
	1	0.16	0.93	0.86	0.78	0.71	0.68	0.59	0.52	0.47
	2	0.71	0.18	0.94	0.86	0.82	0.74	0.67	0.59	0.53
	3	0.67	0.69	0.21	0.92	0.85	0.79	0.71	0.65	0.58
	4	0.62	0.64	0.68	0.22	0.90	0.86	0.78	0.70	0.64
Protein	5	0.59	0.60	0.62	0.69	0.21	0.92	0.85	0.80	0.72
	6	0.51	0.55	0.57	0.63	0.67	0.20	0.91	0.82	0.79
	7	0.46	0.49	0.51	0.56	0.61	0.66	0.15	0.91	0.84
	8	0.40	0.43	0.44	0.51	0.56	0.59	0.67	0.15	0.92
	9	0.35	0.39	0.41	0.46	0.49	0.51	0.59	0.64	0.13
